# Supplementary material for: Serum brain-derived neurotrophic factor levels in type 2 diabetes mellitus patients and its association with cognitive impairment: A meta-analysis
Source: PLoS One. 2024 Apr 22;19(4):e0297785. doi: 10.1371/journal.pone.0297785 (PMC11034647; doi:10.1371/journal.pone.0297785)
Supplement: Attachment — Submitted filename: Response letter.docx [file pone.0297785.s003.docx]

**Response letter to reviewers**

Dear Editors and Reviewers:

Thank you for your letter and for the reviewers’ comments concerning our manuscript entitled “Serum brain-derived neurotrophic factor levels in type 2 diabetes mellitus patients and its association with cognitive impairment: a meta-analysis” (Manuscript Number.: PONE-D-23-23408). Those comments are all valuable and very helpful for revising and improving our paper, as well as the important guiding significance to our research. We have studied the comments carefully and have made corrections which we hope meet with approval. Revised portions are marked in red on the paper. The main corrections in the paper and the responses to the reviewer’s comments are as follows:

Reviewer #1: The authors have discussed "Serum brain-derived neurotrophic factor levels in type 2 diabetes mellitus patients and

its association with cognitive impairment". The methodology is appropriate, and the results have been described in line with the findings. The manuscript does have merit for publication but is not suitable for it in its current state. A thorough proofreading is recommended. The other issues are as follows:

**Response:** Thanks for your time and effort spent reviewing our manuscript. We have checked our manuscript thoroughly and made relevant corrections to improve paper quality.

**Introduction**

"T2DM is the most important risk factor implicated in cognitive deficits, including learning, memory, and processing speed. "Is T2DM the most important risk factor for the cognitive deficit? What about aging and neurodegenerative disorders?

**Response:** Thank you for your comments. “T2DM is the most important risk factor for cognitive deficit except aging and neurodegenerative disorders.” We have made relevant corrections in the Introduction section. (Introduction section, paragraph 1)

The subsection under "Introduction" pointing towards the connection between T2DM and cognitive deficits needs reframing and updating with recent literature. I do not see any literature post-2020 either in the background forming the research question or in the discussion of the results, even though a quick search on Pubmed or Google turns up plenty of recent research.

**Response:** Thank you for your comments. We have added literature post-2020 in the Introduction and Discussion.

**Methods**

I suggest an update of the literature search for the meta-analysis, which is currently up to December 2022.

**Response:** Thank you for your comments. The literature as of October 2022 has been retrieved but no other relevant literature has been published.

Was the study registered on a database (e.g., Prospero)? If yes, it would be better to provide the ID.

**Response:** Thank you for your comments. The study was registered on a database and

the ID was CRD42020211608.

"We applied a search strategy based on the combination of relevant terms. Two independent investigators acquired articles and sequentially screened their titles and abstracts for eligibility. Then, full texts of articles deemed potentially eligible were acquired."

How were the articles acquired and screened? Manually or through some screening tool?

**Response:** Thank you for your comments. A systemic search strategy was used to identify the relevant studies published in PubMed, EMBASE, and the Cochrane Library from inception to December 2022. We applied a search strategy based on the combination of relevant terms. Two independent investigators acquired articles and sequentially screened their titles and abstracts for eligibility. Then, full texts of articles deemed potentially eligible were acquired. Any disagreement would be solved via discussion with the help of a third senior investigator. A screening guide was used to ensure that the selection criteria were constantly applied. The articles were acquired and screened manually.

Inclusion criteria: (1) clinical cross-sectional studies concerning the quantitative values of serum BDNF level in T2DM patients; (2) sufficient data were available for mean and standard deviation analysis of BDNF level; (3) original research. Exclusion criteria: (1) review, abstracts only, letters, comments, guidelines, and case reports; (2) studies in vitro or in animal models; (3) duplicate publications; (4) incomplete data.

" = √( − )/2 [t=tinv (p, Degrees of freedom)]"

The formula is not clear. What is p and what does tinv stand for?

**Response:** Thank you for your comments. p stands for probability and tinv is a function symbol.

**Discussion**

It is unclear whether the authors want to discuss BDNF in relation to T2DM-related cognitive impairment or cognitive impairment in general. The methodology certainly seems to point towards the former, but the "discussion" also has reference to Alzheimer's and "T2DM-comorbid depression", which is confusing. More supporting literature in favor of BDNF and T2DM-related cognitive impairment can be added.

**Response:** Thank you for your comments. We made some changes to the article.
